# Biochemical characterization of the Lassa virus L protein

**DOI:** 10.1074/jbc.RA118.006973

**Published:** 2019-03-29

**Authors:** Dominik Vogel, Maria Rosenthal, Nadja Gogrefe, Sophia Reindl, Stephan Günther

**Affiliations:** From the ‡ Department of Virology, Bernhard Nocht Institute for Tropical Medicine, Bernhard-Nocht-Strasse 74, Hamburg 20359, Germany and; § German Center for Infection Research (DZIF), Partner site Hamburg–Lübeck–Borstel–Riems, Hamburg 20359, Germany

**Keywords:** viral polymerase, recombinant protein expression, viral replication, RNA polymerase, viral transcription, arenavirus, genome replication initiation, hemorrhagic fever, LASV, prime-and-realign mechanism

## Abstract

The L protein of arena- and bunyaviruses is structurally and functionally related to the orthomyxovirus polymerase complex. It plays a central role in the viral life cycle, as it replicates the virus genome and generates viral mRNA via a cap-snatching mechanism. Here, we aimed to biochemically characterize the L protein of Lassa virus, a human-pathogenic arenavirus endemic in West Africa. Full-length 250-kDa L protein was expressed using a baculovirus expression system. A low-resolution structure calculated from small-angle X-ray scattering data revealed a conformation similar to that in the crystal structure of the orthomyxovirus polymerase complex. Although the L protein did not exhibit cap-snatching endonuclease activity, it synthesized RNA *in vitro*. RNA polymerization required manganese rather than magnesium ions, was independent of nucleotide primers, and was inhibited by viral Z protein. Maximum activity was mediated by double-stranded promoter sequences with a minimum length of 17 nucleotides, containing a nontemplated 5′-G overhang, as in the natural genome context, as well as the naturally occurring base mismatches between the complementary promoter strands. Experiments with various short primers revealed the presence of two replication initiation sites at the template strand and evidence for primer translocation as proposed by the prime-and-realign hypothesis. Overall, our findings provide the foundation for a detailed understanding of the mechanistic differences and communalities in the polymerase proteins of segmented negative-strand RNA viruses and for the search for antiviral compounds targeting the RNA polymerase of Lassa virus.

## Introduction

Lassa virus (LASV)^4^ belongs to the Old World clade of the family of Arenaviridae. It is the causative agent of Lassa fever, a potentially fatal viral hemorrhagic fever in humans for which only limited treatment options and no licensed vaccine are available. In 2018, the unusually large seasonal Lassa fever outbreak in Nigeria emphasized the relevance of the virus for public health in West Africa where Lassa fever is endemic (1). The World Health Organization has listed Lassa fever as a priority disease for urgent research and development (http://www.who.int/blueprint/priority-diseases/en/).^5^

The virus particle consists of only four structural proteins and a lipid envelope as well as a segmented single-stranded RNA genome in negative orientation. LASV replicates in the cytoplasm of the host cell. The key player during viral genome replication and transcription is the ∼250-kDa viral L protein. Most data on the L protein were generated using cell-based assays like the LASV minireplicon system. This assay was used to propose a cap-snatching endonuclease in the N-terminal region of the LASV L protein (3), which was subsequently confirmed by X-ray crystallography (4). In addition, minireplicon experiments confirmed an RNA-dependent RNA polymerase (RdRp) domain that had been predicted in the center of the L protein (5, 6) and identified specific amino acid residues in the C-terminal region that are essential for viral transcription but not replication (7). More recently, experiments with recombinant L protein of the New World arenavirus Machupo provided first insight into promoter binding and basic features of RNA synthesis by the protein (8, 9). Machupo virus L protein also possesses terminal transferase activity as described for RNA-dependent RNA polymerases of positive-strand flaviviruses (9, 10).

The viral genome comprises two segments (S and L), whereby each segment encodes for two proteins. The two open reading frames per RNA segment are in ambisense orientation and separated by a highly structured intergenic region (11). Additionally, the 5′-end and 3′-end of each genome segment contain 19 terminal nucleotides (nt) that are highly conserved among the arenaviruses and represent the promoter sequences (12). Due to complementarity of the 5′ and 3′ termini, the promoter presumably forms a double strand. During genome replication and transcription, the L protein synthesizes three distinct RNA species: (i) antigenomic complementary RNA (cRNA), (ii) genomic viral RNA (vRNA), and (iii) capped, nonpolyadenylated viral mRNA. Viral transcription depends on short, capped RNA primers probably derived from cellular mRNAs by a mechanism called cap snatching (13, 14). In contrast, it is assumed that genome replication is initiated *de novo* by a prime-and-realign mechanism similar to the initiation of influenza virus cRNA synthesis and Hantaan virus genome replication (14–17). The following mechanism is hypothesized for initiation of arenavirus RNA replication. An incoming GTP molecule is bound opposite to a C base at position +2 of the template strand and extended to a GC dinucleotide. The latter is realigned to positions −1 and +1 of the template and serves there as primer for elongation. A main argument for this hypothesis has been the existence of a nontemplated G at the 5′-ends of arenavirus vRNA and cRNA that would originate from the translocated dinucleotide (11, 13–15, 18). In an *in vitro* RNA synthesis assay using purified Machupo virus L protein, the observed product was about 1 nucleotide longer than the template, indicating that the L protein and no other viral or cellular protein is responsible for the attachment of the nontemplated G (9).

Mutational analysis of the highly conserved 19-nt promoter sequences using the LASV minireplicon system suggests that positions 1–12 interact in a base-specific manner with the replication complex, whereas at positions 13–19 only the base pairing between 3′ and 5′ termini is important for the function (12). Kranzusch *et al.* (8) demonstrated stronger binding of the 3′-vRNA promoter strand (which is also the template for replication or transcription initiation) compared with 5′-vRNA promoter strand to the Machupo L protein and defined a sequence motif at positions 2–5 of the 3′-vRNA essential for binding to the L protein. Atomic structures of bat influenza A and influenza B virus polymerase complexes as well as La Crosse orthobunyavirus L protein revealed a separate binding pocket for the 5′-promoter strand outside the polymerase active site in a so-called hook conformation (19–21). However, there is no evidence for formation of a 5′-hook structure during arenavirus replication so far.

As the L protein plays a central role during viral replication and transcription, it represents a promising drug target. Although basic enzymatic properties have been described for the L protein of Machupo virus, details of the molecular mechanisms during replication and transcription are still unknown. Here, we present the expression of LASV L protein in insect cells using a baculovirus system, purification of the protein, and establishment and use of *in vitro* assays to investigate L protein functions, specifically mechanistic details of the interaction with the promoter and the replication initiation. The presented experimental systems and data on the recombinant LASV L protein provide a basis for more detailed functional studies as well as high-throughput screening of antiviral compounds targeting the polymerase of LASV in the future.

## Results

### Effect of affinity tags on LASV L protein activity in the minireplicon system

**Figure 1.**
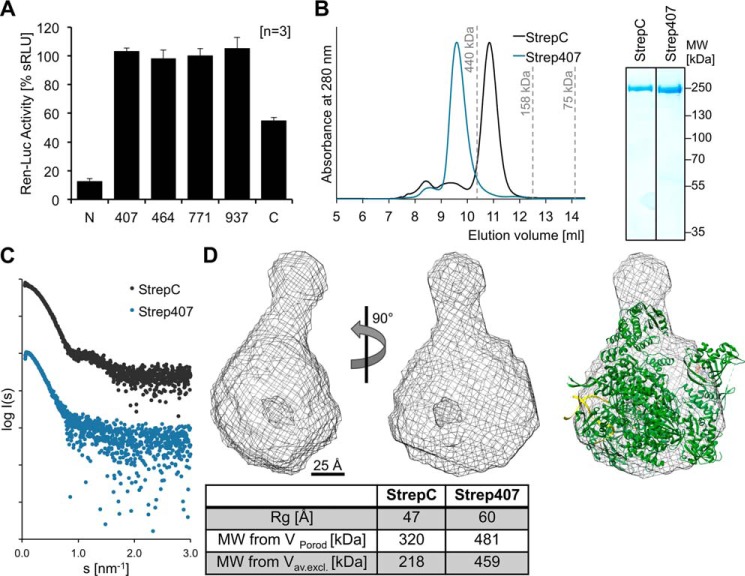
**Influence of affinity tags in varying positions on the activity and structure of the LASV L protein.** *A*, analysis of the influence of a StrepII-tag inserted at the indicated positions on the activity of the LASV L protein using the LASV minireplicon system. The activity of the L protein was measured via Ren-Luc reporter gene expression and is shown as mean of three independent experiments (*n* = 3) in sRLU. *Error bars* represent S.D. sRLU values were log-transformed and then normalized with respect to WT L protein (100%) and negative control (0%). *B*, size-exclusion chromatography and Coomassie-stained SDS-PAGE analysis of purified LASV L protein with C-terminal (StrepC) and internal StrepII-tag after position 407 (Strep407) display the high purity and monodispersity of the L proteins. The Strep407 L protein elutes from the size-exclusion column as a dimer, whereas the StrepC L protein elutes as a monomer. Elution volumes of standard proteins (with sizes of 440, 158, and 75 kDa) for column calibration are indicated. *C*, experimental SAXS scattering profiles of Strep407 (*blue*) and StrepC (*black*) LASV L proteins show the expected conformational differences. *D*, the average SAXS envelope of the C-terminally tagged and monomeric LASV L protein was calculated with DAMMIF and is shown as *mesh* in front view and rotated by 90°. The crystal structure of the bat influenza A virus polymerase complex (PDB code 6EVK) is shown as a cartoon within the SAXS envelope of LASV L protein. A 25-Å *scale bar* is given. The structures were visualized using UCSF Chimera (59). Below the structures, the table compares the size parameters obtained from the SAXS data between StrepC and Strep407 L proteins.

The L protein of LASV has been extensively studied in cell-based minireplicon systems (3, 6, 7, 22, 23). Here, we focus on biochemical characterization of recombinantly expressed LASV L protein (strain Bantou 289). Initially, we still used the LASV minireplicon system to explore in which positions covalent modifications for protein purification (*i.e.* affinity tags) are compatible with L protein function. The standard modifications are small peptides, which are linked to the N or C terminus of the L protein, and it is important to mention that both N- and C-terminal modifications have a serious effect on the functionality of the L protein (Fig. 1*A*). We therefore also included internal insertions in positions, which have been described as flexible interdomain linkers (23). The insertions contained the amino acid sequence of the StrepII-tag binding to the Strep-Tactin matrix. L proteins containing a StrepII-tag at internal positions (after residues 407, 464, 771, and 937, respectively) were as functional as WT protein in the minireplicon system, whereas L protein with a C-terminal tag was about 50% less active (Fig. 1*A*). The N-terminal tag reduced activity even by 90%. Therefore, we generated baculovirus constructs for expression in insect cells of L proteins containing internal tags or the C-terminal tag for purification.

### Expression and low-resolution structure of recombinant LASV L protein

The L proteins with StrepII-tag after residue 407 (L-Strep407) and at the C terminus (L-StrepC) were successfully expressed in insect cells using the EMBacY baculovirus expression system (24, 25). However, recombinant baculoviruses for expression of L proteins with internal StrepII-tag after residues 464, 771, and 937, respectively, could not be produced. The expression level for the C-terminally tagged L protein was considerably higher than for the internally tagged protein. The L proteins were purified by Strep-Tactin affinity chromatography followed by heparin affinity and size-exclusion chromatography. The size-exclusion chromatography also revealed a difference in the oligomerization behavior of both proteins: whereas the C-terminally tagged L protein eluted as monomer, the internally tagged L protein eluted as dimer (Fig. 1*B*).

We used the purely monodisperse and monomeric C-terminally tagged L protein to perform small-angle X-ray scattering (SAXS) experiments (Fig. 1*C*). The SAXS model features a compact core domain with a hollow center, which is decorated with at least one protruding subdomain. The low-resolution structure of the LASV L protein has a similar overall shape as crystal structures of the heterotrimeric influenza polymerase complex (Fig. 1*D*) (20, 21, 26); both also have a similar molecular weight. The influenza virus structure could be superimposed with the SAXS envelope, showing that the size and basic shape of the Lassa L and the influenza polymerase complex are comparable. Due to the presence of flexible protruding domains attached to the polymerase core, which were observed in various positions in individual SAXS models, more detailed structural information cannot be derived from the averaged model calculated from the SAXS data. Nevertheless, the SAXS data suggest a conserved architecture of Lassa and influenza virus polymerase complexes. As the dimer conformation of the internally tagged L protein was not purely monodisperse, we refrained from the calculation of a SAXS envelope to avoid the description of potential artifacts (Fig. 1*C*).

### The L protein is an active polymerase

To characterize the enzymatic properties of L protein, we performed *in vitro* RNA synthesis assays with both L-Strep407 and L-StrepC. The L protein was incubated in the presence of a 19-nt template corresponding to the 3′-promoter strand of the LASV genome and NTPs (including radiolabeled [α-^32^P]GTP). In the standard assay, the complementary 19-nt 5′-promoter strand was added in advance as previous experiments with influenza virus have shown an enhancing effect if both promoter strands are present in the RNA polymerase assay (21). Newly synthesized RNA product was visualized after denaturing PAGE by autoradiography.

**Figure 2.**
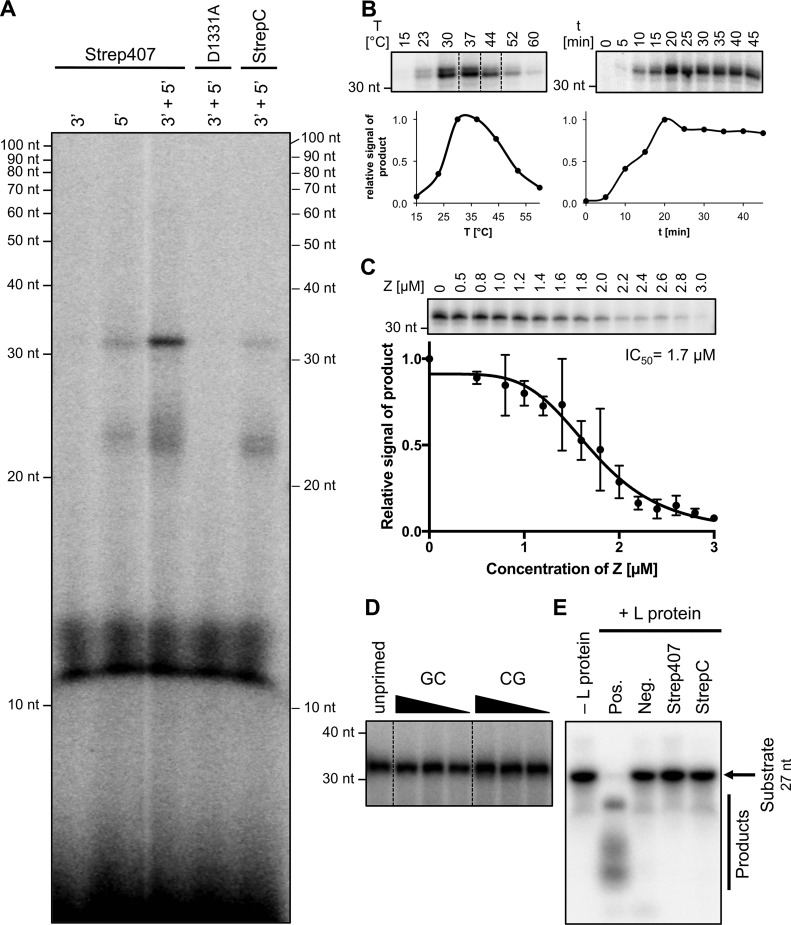
**Enzymatic activities of the Lassa virus L protein.** *A*, only the internally tagged LASV L protein is capable of RNA synthesis in an *in vitro* polymerase assay. Purified LASV L protein with C-terminal tag (StrepC) or internal tag at position 407 (Strep407) or a catalytically inactive mutant of Strep407 (D1331A) were incubated with the conserved 19 nt of the 5′- and/or 3′-genome end (for RNA sequences see Table 1) in the presence of NTPs supplemented with [α-^32^P]GTP for 30 min at 30 °C. Products were separated by denaturing gel electrophoresis and visualized by autoradiography. *B*, RNA synthesis activity of the LASV L protein Strep407 at different temperatures (*left*) and over time (*right*). All lanes included in one *box* originate from the same gel, and *dotted lines* indicate gel cutting for presentation reasons. *C*, inhibition of RNA synthesis activity of the LASV L protein by the LASV Z protein. 1 μm Strep407 L protein was incubated with the indicated amounts of Z protein. For IC_50_ determination, RNA bands were quantified and normalized to the sample without Z protein. The mean and S.D. (*error bars*) of three independent experiments (*n* = 3) is shown and a dose-response curve was fitted to the data. *D*, the RNA synthesis efficiency of the LASV L protein Strep407 is equally high in the presence of only NTPs (unprimed) or with the addition of dinucleotides GC or CG at concentrations from 62.5 to 250 μm. All lanes originate from the same gel, and *dotted lines* indicate gel cutting for presentation reasons. *E*, no RNA cleavage could be observed with the LASV L proteins under the tested conditions. The proteins (0.5 μm L StrepC and Strep407) were incubated with ∼0.3 μm radioactively labeled 27-nt RNA substrate Endo (see Table 1) at 37 °C for 30 min. Substrate and reaction products were separated on a denaturing polyacrylamide gel and visualized by autoradiography. The enzymatically active or inactive isolated cap-snatching endonuclease from Andes hantavirus (2) was used as positive (*Pos.*) or negative control (*Neg.*), respectively.

Pilot experiments confirmed that neither the 3′-vRNA promoter/template strand nor the 5′-vRNA promoter strand alone was sufficient to drive synthesis of clearly detectable amounts of RNA, whereas incubation with both strands resulted in clear signals in the *in vitro* assay (Fig. 2*A*). Consistent with the minireplicon data, the internally tagged L protein was more active than the C-terminally tagged version (Fig. 2*A*, compare *lanes 3′* + *5′ Strep407* and *3′* + *5′ StrepC*). Therefore, L-Strep407 was used in all future experiments. The assay yielded minor reaction products between 20 and 30 nt in length (smear) and a distinct major product of >30 nt. The main product was longer than expected from the length of the template, which presumably is an artifact due to missing termination signals. The assay contains only physically separated short promoter strands rather than the continuous RNA genome comprising all necessary cis-acting signals. An L-Strep407 mutant with exchange of a catalytic residue implicated in RNA polymerization (D1331A) was inactive, demonstrating the specificity of our assay (Fig. 2*A*).

The LASV L protein possesses temperature dependence with an optimum at ∼30–37 °C but remains surprisingly active at temperatures >50 °C (Fig. 2*B*, *left panel*). The main RNA product was already detected after short incubation time (half-maximum after ∼10 min), and saturation was reached after 20 min (Fig. 2*B*, *right panel*). Although template and NTPs were present in excess, a longer incubation time did not result in larger amounts of product. This might indicate that under our reaction conditions the newly synthesized strand is not released from the catalytic site, and thus L protein is blocked for further rounds of polymerization.

Inhibition of L protein activity by the small Z protein has been described for LASV and Machupo virus using the minireplicon system and an *in vitro* assay, respectively (9, 22). We expressed LASV Z protein as described by Hastie *et al.* (27) and purified the monomeric form of the protein (Fig. S4). Using our assay, we confirmed its inhibitory effect on RNA synthesis by the LASV L protein (Fig. 2*C*). Interestingly, the inhibitory effect of Z protein required a specific stoichiometry relative to the L protein. At equimolar concentrations of Z and L protein (1 μm each), almost no inhibition was observed. A reduction in polymerase activity was only observed when the Z protein was present in excess (70% inhibition at 2:1 ratio). LASV Z protein carrying mutation W35A was unable to inhibit polymerase activity (Fig. S5). As this mutation has been described to hamper L–Z interaction (28), our data support the hypothesis that Z protein inhibits polymerase function by direct interaction with the L protein.

Finally, we tested whether the L protein is able to initiate genome replication *de novo* or whether it requires a primer. The RNA synthesis efficiency of L-Strep407 in the absence (unprimed) or presence of GpC or CpG primers was comparable (Fig. 2*D*). Thus, the minimal substrates for initiation of replication and RNA polymerization by L protein are NTPs.

### L protein does not show detectable RNA cleavage activity

Reguera *et al.* (29) studied the isolated LASV cap-snatching endonuclease domain; however, they could not detect RNA cleavage activity in their *in vitro* assays. As the endonuclease may require activation by another region of the L protein, we performed RNA cleavage experiments with L-Strep407 and L-StrepC. However, as with the isolated endonuclease, no decrease in the amount of input RNA under the tested conditions was observed (Fig. 2*E*). A bunyaviral cap-snatching endonuclease was used as a positive control for our assay and showed the expected activity (Fig. 2*E*, *Pos.*).

### Influence of di- and monovalent ions on L protein activity

**Figure 3.**
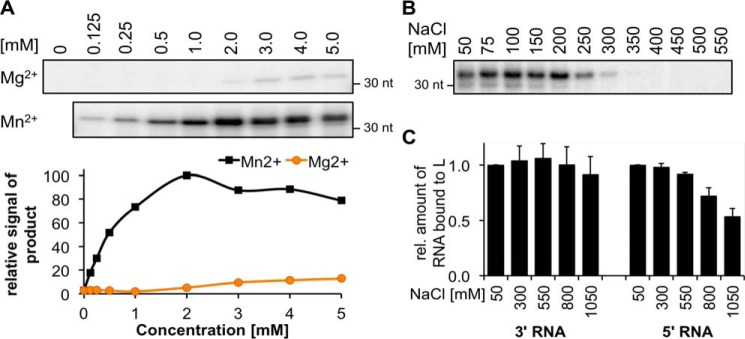
**Influence of metal ions and salt concentrations on the polymerase and RNA binding activities of LASV L protein.** *A*, RNA polymerase activity of 1 μm LASV L protein Strep407 was analyzed in the presence of the indicated concentrations of MgCl_2_ or MnCl_2_. RNA bands were quantified and normalized to the sample with 2 mm MnCl_2_. The experiment was performed as described for the standard polymerase assay. *B*, RNA polymerase activity of 1 μm LASV L protein Strep407 was analyzed in the presence of the indicated concentrations of NaCl, 50 mm KCl, and 2 mm MnCl_2_. The experiment was performed as described for the standard polymerase assay. *C*, RNA binding of LASV L protein Strep407 to the 5′- and 3′-RNA (see Table 1) was determined by an electrophoretic mobility shift assay. 0.6 μm L protein was incubated in a 1:1 ratio with RNA at increasing NaCl concentrations as indicated. The protein–RNA complex was separated from the free RNA by native PAGE, and the relative amount of shifted RNA was determined compared with the sample with the lowest NaCl concentration. Data represent mean of two independent experiments (*n* = 2). *Error bars* represent data range. For an exemplary gel image, see Fig. S2.

As described for other polymerases, RNA synthesis depends on the presence of divalent metal ions (30, 31). The same was observed for the LASV L protein; however, it was much more efficient in the presence of manganese compared with magnesium ions (Fig. 3*A*). The activity plateau was reached at MnCl_2_ concentrations of 2 mm.

To investigate the role of monovalent salt, RNA synthesis activity and promoter binding affinity were measured at different NaCl concentrations. The optimal salt concentration for L protein activity ranged from 50 to 200 mm, which overlaps with the physiological intracellular concentration of monovalent ions (Na^+^ plus K^+^) of ∼150 mm (Fig. 3*B*). The RNA binding ability of L protein was not affected even at NaCl concentrations of 550 mm, indicating robust interaction between RNA and protein (Fig. 3*C*).

### The importance of the 5′-nontemplated G residue for vRNA and cRNA promoter activity

**Figure 4.**
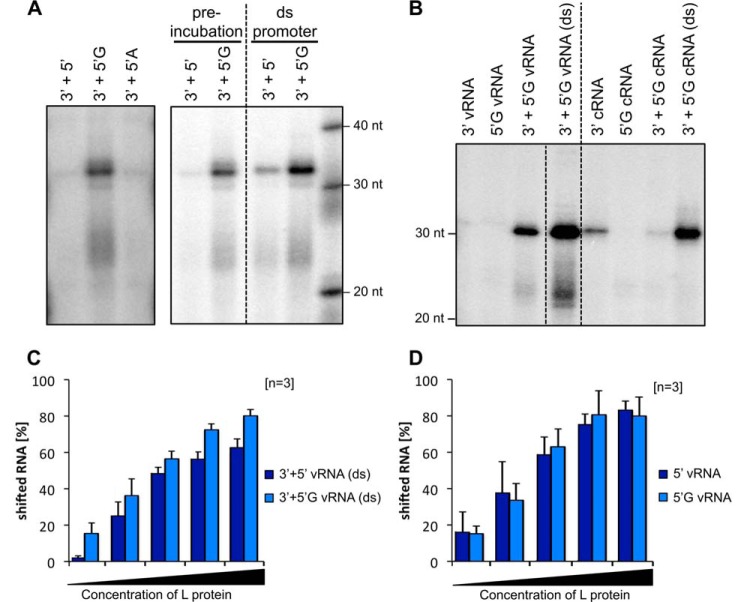
**The 5′-end of the viral promoter activates the polymerase activity of the LASV L protein.** *A*, a single G overhang at the 5′-end is needed for efficient activation of LASV L protein polymerase. 1 μm LASV L protein Strep407 was incubated with 0.6 μm 3′-end of the promoter as template and different variants of the 5′-end: the complementary 5′-end without overhang (*5′*), the 5′-end with a single G overhang (*5′G*) as found on the viral genome, and the 5′-end with a single A overhang (*5′A*) (for RNA sequences see Table 1). Either the L protein was preincubated with the 5′-end for 30 min followed by the addition of the 3′-template, or both RNA molecules were annealed and added together as double-stranded promoter RNA (*ds promoter*). The experiment was performed as described for the standard polymerase assay. All lanes included in one *box* originate from the same gel, and *dotted lines* indicate gel cutting for presentation reasons. *B*, the L protein is not equally active on the vRNA and the cRNA. 5′- and 3′-promoter ends of the vRNA and cRNA (see Table 1) were added to the L protein Strep407 either separately, one after the other (5′-end preincubation for 30 min and then addition of 3′-end), or annealed as dsRNA (*ds*). The experiment was performed as described for the standard polymerase assay. All lanes originate from the same gel, and *dotted lines* indicate gel cutting for presentation reasons. *C* and *D*, binding affinities of the LASV L protein Strep407 to the double-stranded vRNA promoter (*ds*) or the single-stranded 5′-end, respectively, with and without the additional G were analyzed by electrophoretic mobility shift assays. Increasing amounts of L protein (0–1 μm) were incubated with 0.6 μm indicated RNA (see Table 1). The protein–RNA complex was separated from the free RNA by native PAGE, and the relative amount of shifted RNA was determined by quantification. Data represent mean of three independent experiments (*n* = 3). *Error bars* represent S.D. For exemplary gel images, see Fig. S3, *A* and *B*.

Sequencing data showed that the 5′-ends of the arenavirus vRNA and cRNA contain an additional nontemplated G residue including 5′-triphosphate, which is assumed to originate from the prime-and-realign mechanism for replication initiation (14, 15). It was proposed that this nontemplated G, which results in a pppG overhang in the double-stranded promoter, protects this dsRNA portion from recognition by Rig-I (32). Additionally, the prime-and-realign mechanism could play a role in maintaining the integrity of the genome ends (16). Our data show that the G overhang is also important for the enzymatic function of the L protein. If the assay was performed in the presence of a 5′-vRNA promoter strand containing a 5′-nontemplated G, we measured up to 10-fold higher amounts of RNA product compared with the assay with 5′-vRNA without G (Fig. 4*A*).

As mentioned, the standard polymerase assay involves preincubation of the L protein with the 5′-vRNA promoter strand prior to the addition of the 3′-vRNA promoter/template strand and NTPs. As a sequential interaction of L protein with the promoter strands might not reflect the natural situation, we performed assays in the presence of freshly annealed double-stranded promoter RNA. Addition of a preannealed vRNA promoter with and without G overhang further activated the L protein compared with the sequential addition of the promoter ends (Fig. 4, *A*, *right panel*, compare preincubation and ds promoter, and *B*, *left side*, compare *lanes 3′* + *5′G vRNA* with *3′* + *5′G vRNA (ds)*). An even stronger activation was observed with the double-stranded cRNA promoter containing a G overhang (Fig. 4*B*, *right side*, compare *lanes 3′* + *5′G cRNA* with *3′* + *5′G cRNA (ds)*). This suggests that the L protein has an intrinsic mechanism to specifically bind the double-stranded promoter with G overhang, the natural conformation, and then separate both strands for transcription or genome replication initiation. The data also indicate that the vRNA promoter with G overhang mediates stronger polymerase activity compared with the cRNA promoter with G overhang (Fig. 4*B*, compare *left* and *right sides*). This might be associated with the key role of the vRNA promoter in primary transcription of nucleoprotein (NP) and L genes upon infection of a cell.

To test whether the enhanced RNA synthesis activity mediated by the G overhang might be related to increased affinity of the promoter to the L protein, we performed RNA binding experiments. The L protein had somewhat higher affinity to the double-stranded vRNA promoter with G overhang compared with the promoter lacking the overhang (Fig. 4*C*). This effect required a double strand, as binding of just the 5′-vRNA promoter strand was not enhanced by attachment of the nontemplated G (Fig. 4*D*). These data suggest that the G overhang enhances polymerase activity at least in part via enhanced binding of the promoter to the L protein.

To test whether any single-nucleotide overhang or specifically the presence of a G is required for the activation of L protein, we performed the assay in the presence of the 5′-vRNA with an A overhang. The change from G to the chemically similar A abolished the activation, indicating that the effect is base-specific at least among the purine nucleotides (Fig. 4*A*, *left panel*, compare *lanes 3′* + *5′G* with *3′* + *5′A*).

### Evidence for the prime-and-realign mechanism

The prime-and-realign mechanism for *de novo* initiation of replication (14–16) and possibly also transcription (33–35) has been proposed based mainly on sequencing data from a number of segmented negative-strand RNA viruses (sNSVs). To provide biochemical evidence for this mechanism, we performed assays in the presence of various di- and trinucleotide primers. In contrast to the above described experiments, where we added radioactively labeled GTP to visualize the product, we used unlabeled NTPs but end-labeled the di- and trinucleotide primers to specifically visualize the extended primers.

**Figure 5.**
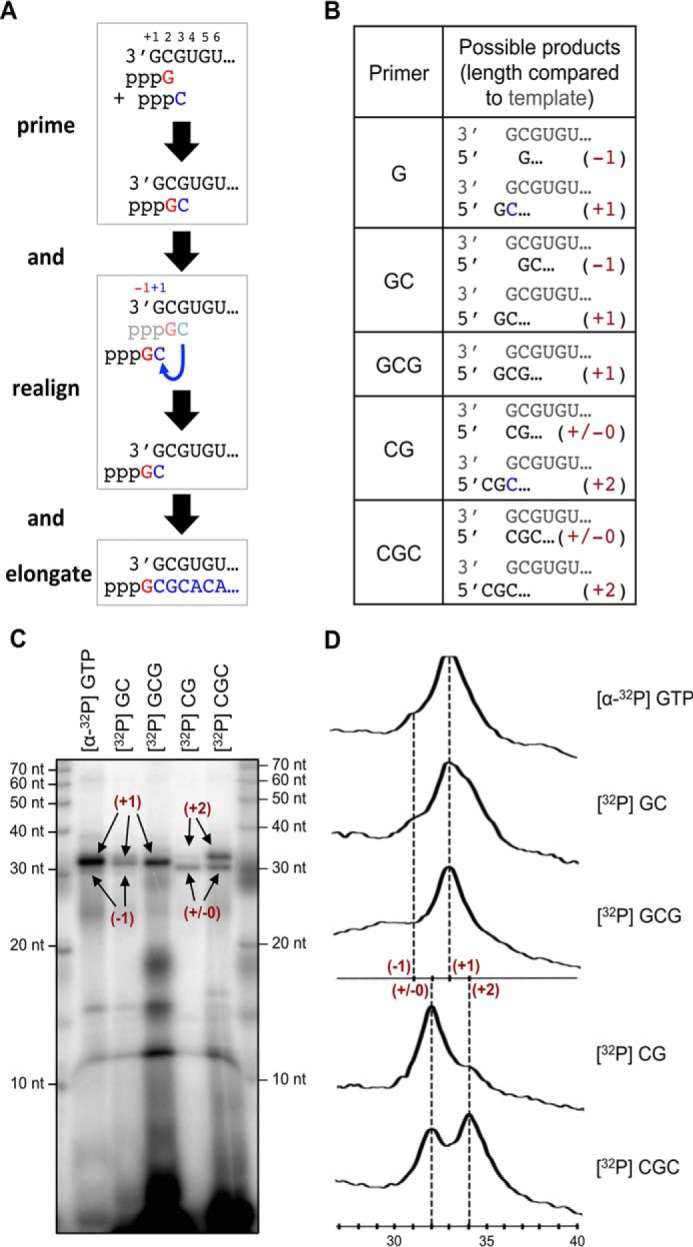
**The prime-and-realign mechanism for initiation of replication.** *A*, schematic representation of the prime-and-realign mechanism. A GTP primes the reaction by binding to the second nucleotide (position +2) of the template. After addition of the next nucleotide, a C, the resulting dinucleotide is not further elongated, but it dissociates, and the C binds to the first G (position +1) of the template sequence in the realign step. This realigned GC dinucleotide is then elongated, which results in an RNA product with 1 additional nucleotide (+1). *B*, the table summarizes the mono-/di-/trinucleotides used in this study as primers and the possible products with and without realignment. In *parentheses*, the relative length of the product RNA is given. *C*, RNA products synthesized by the LASV L protein in the presence of the radioactively labeled primers shown in *B*. The experiment was performed as described for the standard polymerase assay. Where indicated, radioactively labeled primers were used instead of [α-^32^P]GTP. The lengths of the respective products according to *B* are indicated. *D*, the intensity profiles of the gel lanes from *C* were generated using ImageJ software (61) and illustrate the size differences between the product bands shown in *C*. Sizes were determined by linear regression using the RNA marker (*lower x axis*). In *parentheses*, the lengths of the peak products are given according to *B*.

Fig. 5*A* depicts the hypothesis for the prime-and-realign mechanism. Fig. 5*B* shows the primers used in this study along with the products, which would predictably be generated depending on where a primer binds on the template and whether or not it is extended and realigned before elongation. The data are shown in Fig. 5*C* and further analyzed in Fig. 5*D*. Primer GC mimics the dinucleotide that is generated during *de novo* priming. It may hybridize at two sites on the template, namely at positions +2/+3 and −1/+1, corresponding to the proposed binding sites of the *de novo* primed dinucleotide before and after realigning, respectively. Consistent with initiation at these two sites, two products were generated in the polymerase assay, a shorter, very minor RNA species and a longer, major RNA species, both showing a length difference of 2 nucleotides (Fig. 5, *C*, *lane GC*, and *D*). The abundance of the longer RNA species suggests that the −1/+1 site (“realigned primer” positions) is preferred for replication initiation. A similar result was observed with primer CGC. This primer may bind at template positions +1/+2/+3 and −2/-1/+1, respectively. As for primer GC, two RNA species showing a length difference of 2 nucleotides were generated in the polymerase assay, apparently corresponding to initiation at these two sites (Fig. 5, *C*, *lane CGC*, and *D*). As expected from the additional 5′-C residue of primer CGC compared with primer GC, the two RNA species primed by CGC are 1 nucleotide longer than the corresponding RNA species primed by GC. However, in contrast to the GC-primed RNAs, the two CGC-primed species were equally abundant. Primer GCG differs from primers GC and CGC in the way that it may bind only at a single site at the template (−1/+1/+2) that overlaps with the realigned primer position. Accordingly, only a single RNA species was observed in the polymerase assay, corresponding in length to the more abundant RNA species primed by GC (Fig. 5, *C lane GCG*, and *D*). Both predictably share the same 5′-end, *i.e.* the −1 position relative to the template. In line with the prime-and-realign hypothesis, these data indicate that the L protein employs at least two replication initiation sites on the template, one corresponding to the initial binding site of the dinucleotide primer (+2/+3) and one corresponding to the realigned primer position (−1/+1). Whether primers GC and CGC bound first to the +2/+3 site and a fraction was subsequently relocated to the −1/+1 site and/or whether they bound instantly to both sites cannot be inferred from the data.

To provide evidence for a realign scenario, we tested GTP (the presumed natural priming nucleotide) and CG primer. Both GTP and CG may initially only bind to the +2 template position and require extension by a C to generate primers, GC and CGC, that may be relocated and hybridize via the newly added C residue to the +1 template position. GTP priming yielded the same RNA species as GC priming (Fig. 5, *C*, *lanes GTP* and *GC*, and *D*). Similarly, CG priming yielded the same RNA species as CGC priming (Fig. 5, *C lanes CG* and *CGC*, and *D*). This strongly suggests that GTP and CG were indeed extended and realigned as proposed by the prime-and-realign hypothesis. However, GTP primarily yielded RNA supposedly resulting from realignment to the −1/+1 site (product length, +1), whereas CG priming primarily yielded transcripts initiated at the +1/+2 site of the template (product length, ±0). This suggests that relocation of a trinucleotide (CGC) to the −2/-1/+1 site is less efficient than relocation of a dinucleotide (GC) to the −1/+1 site, perhaps due to the higher energy needed to release a trinucleotide from the complementary strand or because of steric constraints at the −1/+1 site.

### Relevance of base pairing in the promoter

**Figure 6.**
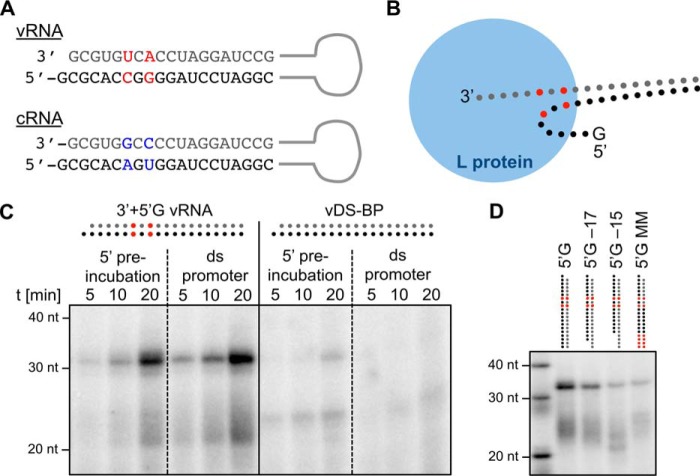
**Arenavirus promoter structure and the importance of mismatched and paired regions.** *A*, promoter sequences of the arenavirus S segment vRNA and cRNA. Mismatched bases of vRNA and cRNA promoter are colored in *red* and *blue*, respectively. *B*, cartoon of the proposed binding mode of the promoter to the L protein, emphasizing the distinct binding sites for the promoter ends. Base mismatches are colored in *red* according to *A. C*, mismatched bases in the early promoter region help the L protein to open the double-stranded promoter. As described before, the LASV L protein Strep407 was either preincubated with the 5′-end of the promoter for 30 min followed by the addition of the 3′-end template, or the annealed promoter was added as dsRNA (*ds promoter*). The native promoter (*left*; depicted according to *A*) was compared with a mutated promoter (*right*) where all 19 nucleotides can form the correct Watson–Crick bp (*vDS-BP*; for RNA sequences see Table 1). *D*, influence of truncations and mismatches in the far end of the vRNA promoter. The 5′-end of the vRNA promoter (*5′G*) was either truncated (*5′G-17* and *5′G-15*), or mismatches were introduced in positions 16–19 (*5′G MM*). The experiment was performed as described for the standard polymerase assay.

The double-stranded arenaviral promoter of the S genome segment contains two mismatched bases at positions +6 and +8 (Fig. 6*A*). To investigate the relevance of this conserved feature, we abolished the mismatches and tested the activity of L protein with all 19 positions of the double-stranded promoter being perfectly base-paired (see Table 1). As above, RNA synthesis was measured both after sequential addition of 5′-vRNA and 3′-vRNA promoter strands and after addition of the preannealed double-stranded vRNA promoter. The mismatched promoter mediated efficient RNA synthesis irrespective of whether it was added sequentially or preannealed (Fig. 6*C*, *left panel*). However, the perfectly matched promoter mediated minimal and no RNA synthesis after sequential addition and preannealing, respectively (Fig. 6*C*, *right panel*). We propose that the mismatches aid the L protein in melting the double-stranded promoter and facilitate the correct positioning of the single-stranded promoter/template for initiation.

**Table 1:** **Synthetic RNA used in the assays** The table lists the RNA oligonucleotides that were used in the described assays. The specific sequence, length in nucleotides, and the identifier used to label the RNA in the experimental descriptions are given. Bold and underlined letters mark nucleotides that have been changed compared with the original sequence. RNAs were synthesized by Biomers.

RNA species	Identifier	Length [nt]	Terminus	Sequence	Terminus
vRNA	5′	19	5′-HO-	CGCACCGGGGAUCCUAGGC	-OH-3′
vRNA	5′G	20	5′-HO-	GCGCACCGGGGAUCCUAGGC	-OH-3′
vRNA	5′A	20	5′-HO-	**A**CGCACCGGGGAUCCUAGGC	-OH-3′
vRNA	5′G-17	18	5′-HO-	GCGCACCGGGGAUCCUAG	-OH-3′
vRNA	5′G-15	16	5′-HO-	GCGCACCGGGGAUCCU	-OH-3′
vRNA	5′G MM	20	5′-HO-	GCGCACCGGGGAUCCU**GAAU**	-OH-3′
vRNA	vDS-BP (5′-end)*^a^*	20	5′-HO-	GCGCAC**A**G**U**GGAUCCUAGGC	-OH-3′
vRNA	vDS-BP (3′-end)*^a^*	19	3′-HO-	GCGUGUCACCUAGGAUCCG	-OH-5′
vRNA	3′	19	3′-HO-	GCGUGUCACCUAGGAUCCG	-OH-5′
cRNA	c5′	19	5′-HO-	CGCACAGUGGAUCCUAGGC	-OH-3′
cRNA	c5′G	20	5′-HO-	GCGCACAGUGGAUCCUAGGC	-OH-3′
cRNA	c3′	19	3′-HO-	GCGUGGCCCCUAGGAUCCG	-OH-5′
Other RNA	Endo	27	5′-HO-	GAUGAUGCUAUCACCGCGCUCGUCGUC	-OH-3′

*^a^* Corresponds to combination of 5′-G cRNA and 3′-vRNA.

Base complementarity in the distal region of the promoter (positions 13–19) has been proposed to be important for promoter strength in the cell-based minireplicon system (12). We measured the RNA synthesis of L protein in the presence of the natural vRNA promoter and promoter versions containing truncations or mismatches in the distal region. The results show that a minimal length of 17 nucleotides is important for L protein function (Fig. 6*D*, *lanes 5′G*, *5′G-17*, and *5′G-15*). The introduction of mismatches at positions 16–19 also reduces RNA yield (Fig. 6*D*, *lane 5′G MM*). These results demonstrate that complementarity in the distal promoter activates RNA synthesis via direct interaction with L protein.

## Discussion

After the determination of the first atomic structures of the polymerase complexes from sNSVs, our understanding of the RNA polymerization mechanism, the initiation of replication and transcription, and the structure of the viral RNA promoter has changed dramatically (20, 21). Still, many open questions remain with one central enigma: how similar are the molecular mechanisms of replication and gene expression between the different viruses of this diverse group? How likely is it that an antiviral compound targeting the RNA polymerase of one virus is also effective on the others? Some striking variations in the replication and transcription mechanisms have already been shown, and many have been proposed (36–40). Most of the atomic structures are from influenza viruses, and nearly all data on other viruses are from cell-based experiments such as minireplicon systems or virus-like particles. For a profound understanding of the molecular mechanisms, comparative studies based on isolated, enzymatically active protein and RNA components are crucial.

This study focused on the L protein of the human-pathogenic LASV. We present an enzymatically active protein with an internal affinity tag, which allows for easy and fast production of high amounts of pure protein. The C-terminally tagged version was hardly active. There is a significant difference in the quaternary conformation between the internally and the C-terminally tagged L protein (dimer *versus* monomer), which might account for higher enzymatic activity of the former compared with the latter conformation. It is conceivable that the native C terminus of L protein, which is only present in the dimer, plays a role in activation of the protein, either directly or via facilitating dimerization. In light of this finding, the interpretation of data produced with terminally modified arenavirus L proteins might need to be revisited. In the following, our data will be discussed in the context of known structural and functional data from other sNSVs.

The overall conformation and shape of the Lassa virus L protein is similar to the known crystal and cryo-EM structures of sNSV polymerases with a compact core domain, a hollow center, and more flexible accessory domains (8, 19–21, 26). In addition, the capability of the L protein to initiate replication *de novo* and its high binding affinity to the viral RNA genome ends have been described for other sNSVs, including influenza and Machupo viruses (8, 19, 21). However, the RNA synthesis by the L protein of Machupo virus and the polymerase complex of influenza virus could be enhanced by dinucleotide primers, which are the first product of the prime-and-realign mechanism (8, 41). The LASV L protein does not show this effect in the presence of comparable concentrations of dinucleotide primers. It might have a higher affinity to GTP, which increases the initiation efficiency so that the formation of the dinucleotide is no longer the rate-limiting step. Furthermore, we provide biochemical evidence for the activation of the L protein by the nontemplated G at the 5′-end of the virus genome and the initiation of replication through the prime-and-realign mechanism. The latter has been hypothesized for a long time and might be a general feature of RNA polymerases from sNSVs (14–17). The small Z protein is known to inhibit arenavirus replication and transcription (22, 42, 43). We confirm that this effect is mediated by LASV L protein in analogy to the data published for the recombinant L protein from Machupo virus (9).

The very N terminus of the arenavirus L protein apparently contains the RNA endonuclease for the cap-snatching mechanism. This conclusion is derived from minireplicon data showing a transcription-deficient phenotype when the central residues of the endonuclease active site are mutated (3, 44). Atomic structures also demonstrate an active-site conformation as found in enzymatically active cap-snatching endonucleases of related viruses (4, 29, 44–46). Surprisingly, no or only low RNA cleavage activity could be shown for the isolated endonuclease domain of arenaviruses (4, 29, 44, 46). In agreement with these data, the full-length LASV L protein does not cleave RNA in our assay. This is in contrast to the cap-snatching endonuclease from influenza virus, which is active as an isolated domain as well as in the context of the whole polymerase complex (45, 47). The missing component, which might “switch on” the endonuclease of LASV, is still unknown and obviously not a part of the L protein.

Finally, we present new features on the mechanism of the LASV L protein, which, to our knowledge, have not been described for RdRps of other sNSVs so far. (I) the LASV L protein synthesizes RNA at a much higher rate in the presence of manganese ions than magnesium ions. Our data do not explain the mechanistic details of this effect, although different scenarios are conceivable. The catalytic center could be more efficient with Mn^2+^ than with Mg^2+^ as it is seen for other metal-dependent enzymes (48, 49). Another explanation could be the presence of an allosteric, structurally stabilizing Mn^2+^-binding site outside the active site, which is not essential for the RNA synthesis but enhancing. And finally, it was shown for hepatitis C virus that Mn^2+^ strongly increases the binding affinity of the polymerase to the first GTP and thereby accelerates the initiation step (50). (II) we could advance our understanding on conserved features of the arenaviral promoter by studying the initial interaction of the polymerase with the promoter in single-stranded *versus* double-stranded conformation, the mismatches in the proximal promoter region (positions 6/8), and the paired bases in the distal promoter region (positions 16/17). And (III) we prove the importance of the overhanging G nucleotide at the 5′-end of the viral RNA for RNA synthesis activity. Taking into account our data and the already published insights from other RdRps, we propose the following model for replication initiation. The L protein initially interacts with the double-stranded promoter, and base complementarity along the 19-nt promoter sequence might be important to stabilize the promoter at this step and facilitate exact positioning of the strands. The mismatches at positions 6 and 8 are important for the subsequent separation of the strands and the binding of the single-stranded promoter ends to their sites (Fig. 6*B*). As hybridization of a vRNA end with the complementary cRNA end would create a perfectly matched promoter, the inactivity of such a promoter might also represent a mechanism to prevent erroneous initiation of replication (Fig. 6*C*). In addition, L protein and NP presumably keep vRNA and cRNA strands well separated to avoid this situation. The G overhang at the 5′-end plays a central role in activating the RdRp. It acts, at least partially, by increasing the binding affinity of the promoter. Whether the additional G is necessary for the formation or stabilization of a 5′-hook structure as found in influenza polymerase complex is still an open question. Following positioning of the template and activation of the enzyme via the 5′-promoter strand, replication is initiated *de novo* by GTP according to the prime-and-realign mechanism as described above.

The *in vitro* system used in this study is extremely valuable for mechanistic studies on the enzymatic properties of the L protein. It is, however, artificial and does not represent the situation as present in an infected cell. We encountered a phenomenon, which is difficult to explain and most likely the consequence of our experimental setup: the product we detected is longer than expected by ∼14 nucleotides. We may only speculate how this product is formed, but we assume that it is a result of using only short promoter fragments missing cis-acting termination signals supposedly present on the full-length genome. In contrast, the short template facilitated the investigation of small length differences of the produced RNA. When using a longer template RNA with a hairpin structure, where the promoter ends are connected by a short RNA sequence, the product had the expected length, but the resolution of the assay was not sufficient to distinguish between single-nucleotide differences (Fig. S1).

In summary, we present new data on the L protein of the highly pathogenic LASV, a protein with a central role in the viral life cycle and a potential drug target. The established protocol for expression and purification of high and pure amounts of the L protein sets the basis for screening experiments with the isolated protein for the development of antiviral drugs for Lassa fever without the requirement for high-containment facilities. As shown by Reich *et al.* (41) for influenza virus, the presented RNA synthesis assay may be transformed to a fluorescence-based assay for high-throughput applications. More biochemical data on L proteins from a diverse set of sNSVs are needed for a better understanding of common and diverse features of their RdRps and how to approach the challenging development of broad-spectrum antiviral agents targeting this large group of human pathogens.

## Experimental procedures

### Cloning, expression, and purification of LASV L protein

The L gene of Lassa virus Bantou 289 (GenBank^TM^ accession number MK044799) with a StrepII-tag at the respective internal or C-terminal position was amplified via PCR. In the same step, primers were used for the introduction of mutations to the gene in case of the catalytically inactive L. Amplified genes were cloned into an altered pFastBacHT B vector using the In-Fusion HD EcoDry Cloning kit (Clontech). After transformation of DH10EMBacY *Escherichia coli* cells (24, 25), which contain a bacmid as well as a plasmid coding for a topoisomerase, with the pFastBac plasmids, recombinant bacmids were isolated and transfected into Sf21 insect cells for recombinant baculovirus production. Hi5 insect cells were used for the expression of the StrepII-tagged L proteins. The harvested cells were resuspended in Buffer A (50 mm HEPES(NaOH), pH 7.0, 1 m NaCl, 10% (w/w) glycerol, and 2 mm DTT) supplemented with 0.05% (v/v) Tween 20 and protease inhibitors (Roche Applied Science, cOmplete mini), lysed by sonication, and centrifuged two times at 20,000 × *g* for 30 min at 4 °C. Soluble protein was loaded on Strep-TactinXT beads (IBA) and eluted with 50 mm biotin (Applichem) in Buffer B (50 mm HEPES(NaOH), pH 7.0, 500 mm NaCl, 10% (w/w) glycerol, and 2 mm DTT). L protein–containing fractions were pooled and diluted with an equal volume of Buffer C (20 mm HEPES(NaOH), pH 7.0) before loading on a heparin column (HiTrap Heparin HP, GE Healthcare). Proteins were eluted with Buffer A and concentrated using centrifugal filter units (Amicon Ultra, 100,000 molecular weight cutoff). The proteins were either used for biochemical assays or further purified by size-exclusion chromatography (SD200, GE Healthcare) in Buffer B for SAXS experiments. Pure L proteins were concentrated as described above, flash frozen, and stored at −80 °C.

### Cloning, expression, and purification of LASV Z

The Z gene of Lassa strain AV was cloned into a pOPINF or pOPINJ vector (51) using the In-Fusion HD EcoDry Cloning kit. The Z protein with N-terminal His_6_ fusion tag (pOPINF) or N-terminal GST fusion protein (pOPINJ) was expressed in *E. coli* strain BL21 Gold (DE3) (Novagen) at 17 °C overnight using terrific broth medium (supplemented with 100 μm ZnSO_4_) and 0.5 mm isopropyl β-d-thiogalactopyranoside for induction. For the Z protein with N-terminal His_6_ fusion tag, after pelleting, the cells were resuspended in Buffer D (50 mm Tris(HCl), pH 8.0, 300 mm NaCl, and 5% (w/w) glycerol) supplemented with 10 mm imidazole, 2.5 mm phenylmethylsulfonyl fluoride, 0.05% (v/v) Triton X-100, and 0.025% (w/v) lysozyme; disrupted by sonication; and centrifuged at 30,000 × *g* for 30 min at 4 °C. The soluble protein was loaded on nickel-nitrilotriacetic acid (GE Healthcare), washed with Buffer D supplemented with 50 mm imidazole, and eluted with Buffer D supplemented with 500 mm imidazole. The protein was dialyzed against Buffer E (50 mm Tris(HCl), pH 7.5, 150 mm NaCl, and 5% (w/w) glycerol), and in the same step the His_6_ tag was removed overnight at 4 °C with GST-tagged 3C protease. GST-tagged 3C protease was removed by incubation with GSH-Sepharose (GE Healthcare), the solution was diluted with an equal volume of Buffer F (20 mm Tris(HCl), pH 7.5), and passed over a heparin column (HiTrap Heparin HP, GE Healthcare) to reduce the amount of oligomeric Z. The pH of the flow-through was adjusted to pH 6.8, again loaded onto a heparin column, and eluted with 1 m NaCl in Buffer F. The protein was further purified by size-exclusion chromatography on an SD200 column (GE Healthcare) in Buffer E. Fractions containing monomeric Z protein were concentrated using centrifugal filter units (Amicon Ultra, 3000 molecular weight cutoff) and used for SAXS measurements. For the Z proteins expressed as GST fusion protein (WT or mutant W35A), purification was done essentially as described by Hastie *et al.* (27). Briefly, after expression, harvesting, and lysis as described above, protein was loaded onto GSH-Sepharose beads. GST fusion tag was cleaved on-column by incubation with GST-tagged 3C protease at 4 °C overnight. Cleaved Z protein was diluted with an equal volume of 20 mm Tris(HCl), pH 8.5, and subjected to anion-exchange chromatography using a HiTrap Q FF column (GE Healthcare). Z protein present in the flow-through was concentrated and further purified by size-exclusion chromatography as described above. Fractions containing monomeric Z protein were concentrated as described above, flash frozen in liquid nitrogen, and stored at −80 °C.

### LASV minireplicon system

The experiments were performed in the context of the T7 RNA polymerase–based Lassa virus minireplicon system essentially as described previously (3, 22). L genes with or without affinity tags at different positions (N-terminal and internal, StrepII; C-terminal, StrepII-His_6_) were cloned into vector pCITE2a. BSR-T7/5 cells stably expressing T7 RNA polymerase (4) were transfected with 250 ng of minigenome expressing *Renilla* luciferase (Ren-Luc), 500 ng of L gene plasmid, 250 ng of pCITE-NP expressing NP, and 10 ng of pCITE-FF-luc expressing firefly luciferase as an internal transfection control per well of a 24-well plate. One day after transfection, cells were lysed in 100 μl of passive lysis buffer (Promega)/well and assayed for firefly luciferase and Ren-Luc activity using the Dual-Luciferase reporter assay system (Promega). Ren-Luc levels were corrected with the firefly luciferase levels (resulting in standardized relative light units (sRLU)) to compensate for differences in transfection efficiency or cell density. sRLU values were log-transformed and then normalized with respect to WT (100%) and negative control (neg; 0%) as follows: log activity of mutant = (log(sRLU_mutant_) − log(sRLU_neg_))/(log(sRLU_WT_) − log(sRLU_neg_)).

### Small-angle X-ray scattering

For L proteins, SAXS was performed after a final size-exclusion chromatography in Buffer B for protein samples with different concentrations (0.5–1.5 mg/ml). Data were collected at the SAXS beamline P12 of PETRA III storage ring of Deutsches Elektronen-Synchrotron (DESY), Hamburg, Germany (52) using a PILATUS 2 M pixel detector at 3.1-m sample distance and 10-keV energy (λ = 1.24 Å); a momentum transfer range of 0.01 Å^−1^ < *s* < 0.45 Å^−1^ was covered (*s* = 4π sinθ/λ where 2θ is the scattering angle). Data were analyzed using the ATSAS 2.6 package (53). The forward scattering, *I*(0), and the radius of gyration, *R_g_*, were extracted from the Guinier approximation calculated with the AutoRG function within PRIMUS (54). GNOM (55) provided the pair distribution function *P*(*r*) of the particle, the maximum size *D*_max_, and the Porod volume. *Ab initio* reconstructions were generated with DAMMIF (56). Twenty independent DAMMIF runs were superimposed by SUPCOMB (57) and averaged using DAMAVER (58). The average excluded volume was extracted from the final Protein Data Bank (PDB) file. Structures were visualized using UCSF Chimera (59). For Z protein, SAXS measurements were performed after a final size-exclusion chromatography in 50 mm Tris(HCl), pH 7.5, 150 mm NaCl, and 5% (w/w) glycerol as described above. Data were analyzed using the ATSAS 2.8 package (60), providing an estimation of the molecular weight based on different algorithms implemented in the program package.

### Electrophoretic mobility shift assay

RNAs (see Table 1) were chemically synthesized (Biomers) and labeled with T4 polynucleotide kinase (New England Biolabs) and [γ-^32^P]ATP (Hartmann Analytic). Labeled RNA substrates were subsequently purified with a Microspin G25 column (GE Healthcare). After heating complementary ssRNA strands in a 1:1 ratio for 5 min up to 95 °C and slowly cooling down to 8 °C over 1 h, dsRNAs were obtained. Reactions containing 0–10 pmol of L protein and 6 pmol of labeled ssRNA or dsRNA were set up in 10 μl of binding buffer (100 mm HEPES(NaOH), pH 7.0, 150 mm NaCl, 50 mm KCl, 4 mm MgCl_2_, 1 mm DTT, 10% glycerol, 0.5 μg/μl poly(C) RNA (Sigma), 0.5 μg/μl BSA, and 0.5 unit/μl RNasin (Promega)) and incubated at 20 °C for 30 min. Products were separated by native gel electrophoresis using 4% polyacrylamide Tris borate–EDTA gels and 0.5-fold Tris borate buffer on ice. Signals were visualized by phosphor screen autoradiography using a Typhoon scanner (GE Healthcare) and quantified if necessary using ImageJ software (61).

### Endonuclease assay

An RNA 27-mer (Endo; see Table 1) was chemically synthesized (Biomers) and labeled with T4 polynucleotide kinase as above. RNA substrates were subsequently purified with a Microspin G25 column. Reactions containing 5 pmol of protein and 3 pmol of labeled RNA were carried out in a volume of 10 μl with 0.5 unit/μl RNasin, 100 mm HEPES(NaOH), pH 7.0, 100 mm NaCl, 50 mm KCl, 2 mm MnCl_2_, 1 mm DTT, and 0.1 μg/μl BSA and incubated at 37 °C for 30 min. The reaction was stopped by adding an equivalent volume of RNA loading buffer (98% formamide, 18 mm EDTA, 0.025 mm SDS, xylene cyanol, and bromphenol blue) and heating the samples at 98 °C for 5 min. Products were separated by electrophoresis on denaturing 7 m urea, 20% polyacrylamide Tris borate–EDTA gels and 0.5-fold Tris borate buffer. Signals were visualized by phosphor screen autoradiography using a Typhoon scanner.

### Polymerase assay

If not indicated otherwise 10 pmol of L protein was preincubated for 30 min with 6 pmol of single-stranded 5′-promoter RNA (see Table 1) in 10 μl of assay buffer (100 mm HEPES(NaOH), pH 7.0, 100 mm NaCl, 50 mm KCl, 2 mm MnCl_2_, 0.5 unit/μl RNasin, 2 mm DTT, and 0.1 μg/μl BSA) before 6 pmol of single-stranded 3′-promoter RNA (see Table 1) and NTPs (0.8 mm UTP/ATP/CTP and 0.5 mm GTP supplemented with 5 μCi of [α-^32^P]GTP) were added in a final reaction volume of 10 μl. In cases indicated, 6 pmol of dsRNA was added together with the NTPs. After incubation at 30 °C for 45 min, the reaction was stopped by adding an equivalent volume of RNA loading buffer and heating the sample at 98 °C for 5 min. Products were separated by native gel electrophoresis using 4% polyacrylamide Tris borate–EDTA gels and 0.5-fold Tris borate running buffer. Signals were visualized by phosphor screen autoradiography using a Typhoon scanner and quantified if necessary using ImageJ software (61).

## Author contributions

D. V., M. R., S. R., and S. G. conceptualization; D. V., M. R., S. R., and S. G. formal analysis; D. V., M. R., and N. G. investigation; D. V., M. R., and S. R. visualization; D. V., M. R., and S. R. writing-original draft; D. V., M. R., S. R., and S. G. writing-review and editing; M. R., S. R., and S. G. supervision; S. G. resources.

## Supplementary Material

Supporting Information

## Footnotes

5 Please note that the JBC is not responsible for the long-term archiving and maintenance of this site or any other third party-hosted site.

## Footnotes

4 The abbreviations used are:

LASV: Lassa virus
RdRp: RNA-dependent RNA polymerase
nt: nucleotides
cRNA: complementary RNA
vRNA: viral RNA
SAXS: small-angle X-ray scattering
dsRNA: double-stranded RNA
NP: nucleoprotein
sNSV: segmented negative-strand RNA virus
Ren-Luc: *Renilla* luciferase
sRLU: standardized relative light units
ssRNA: single-stranded RNA.
