# Supplementary material for: Biochemical characterization of the Lassa virus L protein
Source: J Biol Chem. 2019 Mar 29;294(20):8088–100. doi: 10.1074/jbc.RA118.006973 (PMC6527160; doi:10.1074/jbc.RA118.006973)
Supplement: Supporting Information [file supp_294_20_8088__index.html]

Biochemical characterization of the Lassa virus L protein — Biochemical studies on the Lassa virus L protein — Biochemical characterization of the Lassa virus L protein — Biochemical studies on the Lassa virus L protein — Supporting Information 

# Biochemical characterization of the Lassa virus L protein

## Supporting Information

- Supporting Information (to be published online) - Supporting information, revised
