# Supplementary material for: Biochemical characterization of the Lassa virus L protein
Source: J Biol Chem. 2019 Mar 29;294(20):8088–100. doi: 10.1074/jbc.RA118.006973 (PMC6527160; doi:10.1074/jbc.RA118.006973)
Supplement: Supporting Information [file supp_RA118.006973_142331_1_supp_307471_pp2wq1.pdf]

SUPPORTING INFORMATION

Biochemical characterization of the Lassa virus L protein

Dominik Vogel[1,#], Maria Rosenthal[1,#], Nadja Gogrefe[1], Sophia Reindl[1,]*, Stephan Günther[1,2,]*

Material included: Figure S1
Figure S2
Figure S3
Figure S4
Figure S5

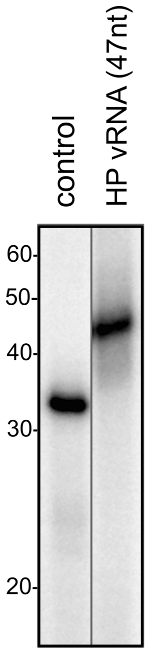

length [nt]	identifier		sequence	
47	HP vRNA	5' HO-	GCGCACCGGGGAUCCUAGGCAUUUUAUAGCCUAGGAUCCACUGUGCG	-OH 3'

Figure S1. A hairpin RNA template leads to a product of the expected length. Purified LASV L protein Strep407 was either pre-incubated with the standard vRNA template used throughout the study, i.e. the conserved 19-nt 5'-end with an additional 5'G residue, followed by addition of the 19-nt 3' genome end (control), or a 47-nt hairpin RNA template containing the terminal 19 nt of 3' and 5' vRNA ends including the additional 5'G residue connected by a linker sequence (HP vRNA). Reactions were carried out in presence of NTPs supplemented with $[\alpha]^{32}$P-GTP for 30 min at 30°C. Products were separated by denaturing gel electrophoresis and visualized by autoradiography. Both lanes originate from the same gel and have been cut for presentation reasons.

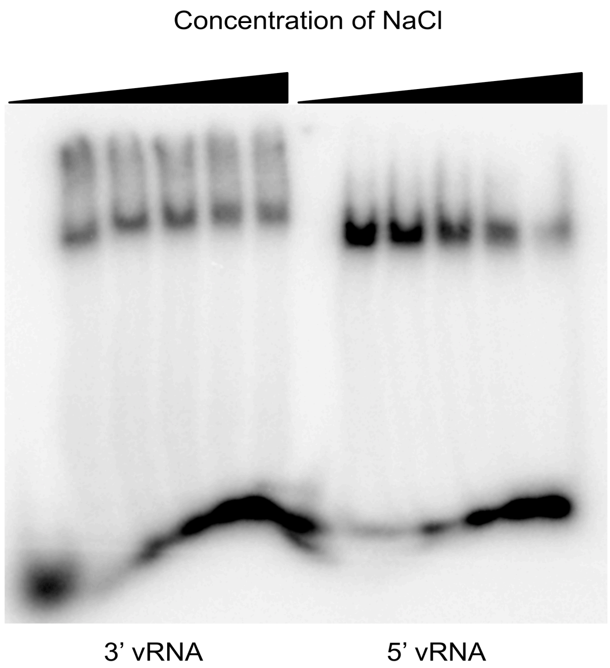

Figure S2. Exemplary gel image for Figure 3C.
RNA binding of LASV L protein Strep407 to the 5' and 3' RNA (see Tab. 1) was determined by an electrophoretic mobility shift assay (EMSA). 0.6 µM of L protein was incubated in a 1:1 ratio with RNA at increasing NaCl concentrations (50 to 1050 mM). The protein-RNA complex was separated from the free RNA by native PAGE and visualized by phosphor screen autoradiography using a Typhoon scanner (GE Healthcare).

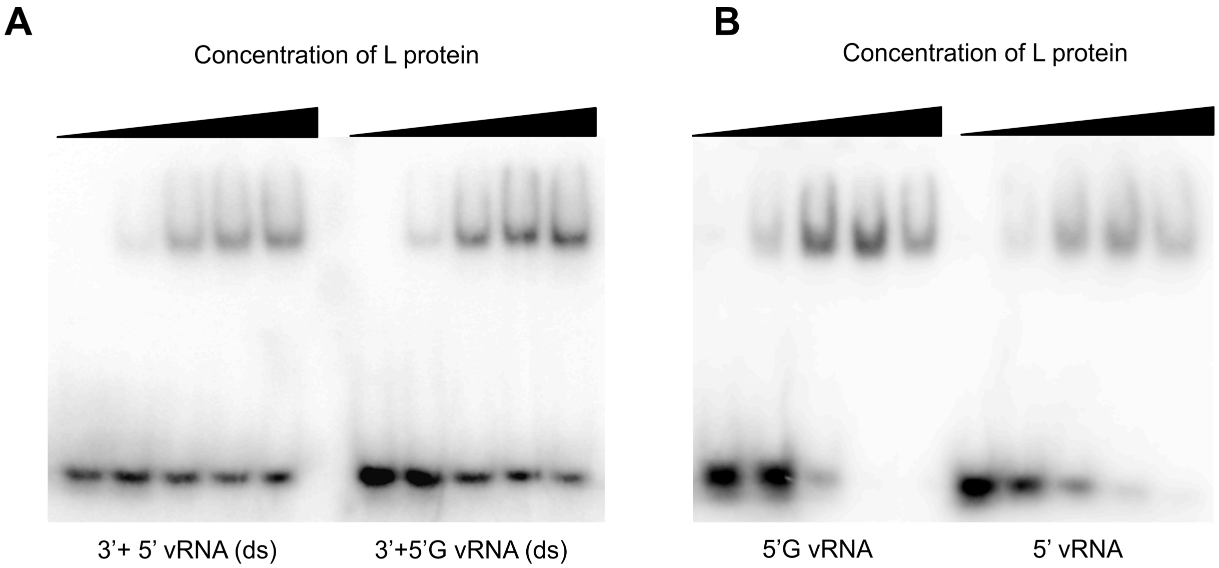

Figure S3. Exemplary gel image for Figures 4C and D.
Binding affinities of the LASV L protein Strep407 to **(A)** the double stranded vRNA promoter (ds) or **(B)** the single stranded 5’-end, respectively, with and without the additional G were analyzed by EMSAs. Increasing amounts of L protein (0 to 1 µM) were incubated with 0.6 µM of the indicated RNA (see Tab. 1). The protein-RNA complex was separated from the free RNA by native PAGE and visualized by phosphor screen autoradiography using a Typhoon scanner (GE Healthcare).

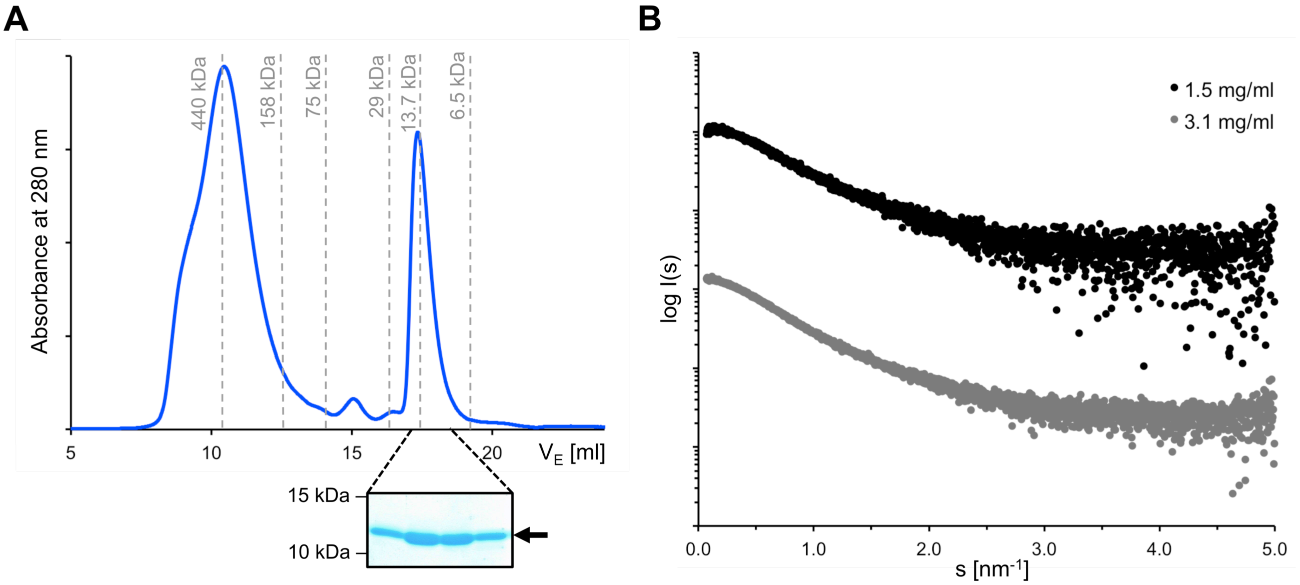

C

concentration of Z protein		estimated MW [kDa]
SEC peak fraction	~ 350 µg/ml	15.6
SAXS	1.5 mg/ml	10.4
	3.1 mg/ml	14.1
**highest concentration of Z protein used in the assay: ~ 32 µg/ml (3 µM)**		

Figure S4. Confirmation of the monomeric state of LASV Z protein.
(A) Size exclusion chromatography (SEC) of LASV Z protein displays two large peaks, representing monomeric and dodecameric Z protein, respectively. Coomassie stained SDS-PAGE analysis of fractions of the second large peak prove the presence of monomeric Z protein. Elution volumes of standard proteins (with sizes between 6.5 and 440 kDa) for column calibration are indicated. **(B)** Experimental SAXS scattering profiles of LASV Z protein at 1.5 mg/ml (black) and 3.1 mg/ml (grey) are presented. **(C)** Comparison of molecular weight estimations of the Z protein extracted from SEC and SAXS measurements at different concentrations with the concentration of Z protein used in the polymerase assay.

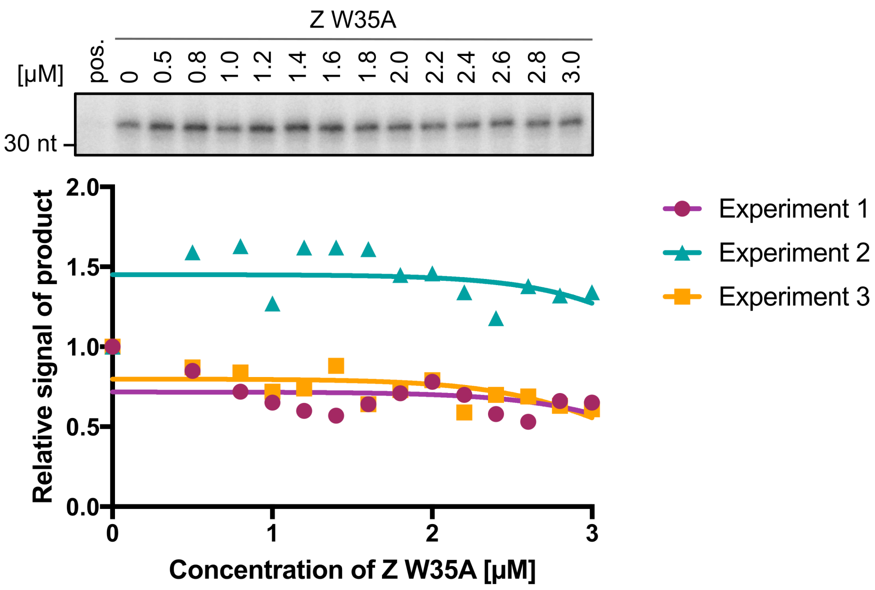

Figure S5. Test of the inhibitory effect of Z protein mutant W35A on L protein activity. Inhibition of RNA synthesis activity of the LASV L protein by the LASV Z W35A protein. 1 µM of Strep407 L protein was incubated with 3 µM of wild-type Z protein (pos.), or the indicated amounts of Z W35A. RNA bands were quantified and normalized to the sample without Z protein. Individual experiments are shown, for each of which a dose response curve was fitted to the data. The parallel shift of the single curves is due to regular experimental variation in the first data point (sample without Z protein).
